# Computed Tomographic Evaluation of Congenital Left Ventricular Outflow Obstruction

**DOI:** 10.2174/1573403X19666230525144602

**Published:** 2023-10-02

**Authors:** Parveen Kumar, Mona Bhatia, Natisha Arora

**Affiliations:** 1 Department of Radiodiagnosis & Imaging, Fortis Escort Heart Institute, New Delhi, India;; 2 Convener, Cardiac Imaging, Cardiological Society of India, Kolkata, 700054, India

**Keywords:** left ventricular outflow obstruction, congenital heart disease, computed tomography, coarctation of the aorta, aortic stenosis, transthoracic echocardiography

## Abstract

Congenital left ventricular outflow obstruction represents a multilevel obstruction with several morphological forms. It can involve the subvalvular, valvar, or supravalvular portion of the aortic valve complex, and may coexist. Computed tomography (CT) plays an important supplementary role in the evaluation of patients with congenital LVOT obstruction. Unlike transthoracic echocardiography and cardiovascular magnetic resonance (CMR) imaging, it is not bounded by a small acoustic window, needs for anaesthesia or sedation, and metallic devices. Current generations of CT scanners with excellent spatial and temporal resolution, high pitch scanning, wide detector system, dose reduction algorithms, and advanced 3-dimensional postprocessing techniques provide a high-quality alternative to CMR or diagnostic cardiac catheterization. Radiologists performing CT in young children should be familiar with the advantages and disadvantages of CT and with the typical morphological imaging features of congenital left ventricular outflow obstruction.

## 1 INTRODUCTION

Left ventricular outflow obstruction comprises a spectrum of stenotic lesions starting from the left ventricular outflow tract (LVOT) and stretching up to the descending portion of the aortic arch. The obstruction can occur at any level distal to the left ventricular (LV) cavity including the LVOT (a portion of LV just beneath the aortic valve), aortic valve, supravalvular region, or more distal aortic segment. There are four broad categories of LV outflow obstructive lesions including subvalvular aortic stenosis, valvular aortic stenosis, supravalvular aortic stenosis, and aortic coarctation [1]. All these lesions increase the LV afterload, resulting in LV hypertrophy and eventually LV failure if left untreated. Transthoracic echocardiography (TTE) is typically the initial imaging modality; however, further cross-sectional imaging using computed tomographic angiography (CTA), cardiovascular magnetic resonance imaging (CMR), and catheter angiography is desired to obtain detailed operative roadmap [2]. The current review highlights the role of CT in the diagnosis, surgical planning, and follow-up of various LVOT obstructive lesions.

## 2 OVERVIEW OF THE MODALITIES

The main diagnostic imaging modalities in assessing congenital left ventricular outflow obstruction include TTE, CTA, CMR, and invasive angiography. Each modality has its own strength and weakness. TTE remains the first-line modality of choice due to its wide accessibility, portability, low cost, lack of radiation exposure, and extensive user experience. TTE has excellent blood tissue contrast and temporal, and spatial resolution. The parasternal long-axis view provides a good assessment of the LVOT. Additionally, the continuous and pulse wave doppler can quantify the severity of various stenotic and regurgitant lesions, which is comparable with catheterization. However, the main downside of this modality is the poor acoustic window in some patients. Besides this, the assessment of aberrant coronary artery anatomy and the extent and severity of the collateral circulation (*i.e*. in aortic coarctation) may also be difficult to evaluate [3].

CMR yields a wealth of anatomic, functional, and hemodynamic information in LVOT defects. Cine imaging through the long axis of the LVOT in 2 orthogonal planes provides a good assessment of the subvalvular, valvular, and supravalvular regions. The information derived from CMR phase contrast (PC) flow velocity mapping can be used to calculate the pressure gradients across the stenotic lesions and the degree of aortic valvular regurgitation. Four-dimensional PC-CMR can detect the abnormal flow patterns which contribute to progressive root dilatation even in patients with non-obstructed bicuspid aortic valves [4, 5]. LV fibrosis is described in patients with severe congenital aortic stenosis. CMR can detect focal LV fibrosis using late gadolinium enhancement; and diffuse fibrosis using T1 relaxometry techniques, commonly referred to as T1 mapping [6, 7]. The main disadvantage of CMR is the long acquisition timings requiring anaesthesia and lengthy sedation in infants and small children [8]. Other disadvantages include poor evaluation of lungs and airways, artefacts from non-compatible implants (*i.e*. prosthetic valves), high cost, and limited availability especially in developing and underdeveloped countries [9]. Additionally, the risk of anaesthesia and emerging risks of gadolinium deposition in the central nervous system and other organs have prompted an FDA advisory on gadolinium contrast, making MRI a less benign imaging option [10, 11]. Cardiac catheterization is usually reserved for patients who are suitable candidates for balloon valvuloplasty, transcatheter valve replacement, or interventions on the right ventricular outflow tract after the Ross operation. If cardiac catheterization is performed, the aortic valve area can be estimated using the Gorlin equation [12]. Aortic regurgitation severity can be evaluated with aortography. The main limitation of catheterization includes invasiveness, prolonged sedation-anaesthesia, and various catheter-related complications.

Multi-detector computed tomography (MDCT) has evolved as a reliable tool for pediatric patients with congenital heart diseases (CHD). The advantages of MDCT include greater accessibility, relatively reduced cost, and superior evaluation of lung and airway abnormalities that may occur concomitantly with complex CHD. The introduction of helical prospective ECG-triggered acquisition at a high pitch [3, 4] has enabled gapless volume data acquisition in one cardiac cycle [13]. There is no need for sedation or anaesthesia with current-generation CT scanners. It is an alternative imaging tool in pediatric patients with contraindications to MR scanning *i.e.* internal cardiac defibrillator, pacemaker, or aneurismal clip. While cardiac valve prostheses, surgical clips, and occlusion devices are not contraindicated for CMR, MDCT is preferable because these implants limit the image quality of CMR. CT offers an exquisite delineation of the LVOT, aortic valve complex, coronary arteries, and ascending aorta. Noncontrast images can be used to identify the calcium deposits within the aortic leaflets and the aorta. The retrospectively gated CT provides multiphase acquisition which is useful in ventricular volumetry. This is useful, especially in patients with contraindications to CMR. The main limitations of CT are iodinated contrast administration and radiation exposure. However, the latest generation scanners using dual source and wide detector technology have dramatically lowered the radiation exposure and sub-millisievert scans are achievable in many children. The reduction in the scan time has also reduced the amount of contrast media reducing the risk of contrast-induced nephropathy [14]. Another limitation of CT includes relatively poor temporal resolution (135 ms with single source scanners), in comparison to angiography (1-10 milliseconds) or MRI (20-50 ms) which are performed without β-blockers. Modern scanners using fast gantry rotation (0.3-0.4 sec), half scan reconstruction, and optional multisector reconstruction have beaten this challenge and can provide excellent temporal resolution (50-65 ms) [15].

Sixty-four-section multidetector CT is the minimum requirement for obtaining optimum quality diagnostic images. There are two acquisition modes: ECG-gated and non-ECG gated. ECG-gated acquisition modes can be prospective or retrospective. The main difference between prospective and retrospective modes is the timing of data acquisition with respect to the cardiac cycle. In the prospective mode, the data is acquired in a predefined phase of the cardiac cycle (*i.e.*, end-systole [30%-40%] or end-diastole [70%-80%]); while in the retrospective mode, the data acquisition is continuous throughout the cardiac cycle (0%-100%). The main advantage of the prospective ECG-gated protocol is lower radiation dose, as X-ray exposure occurs during the selected cardiac phase only. Recently, iterative reconstruction has emerged as a new method of image reconstruction. This technique enables reconstructing low-noise images from intrinsically noisy data, preserving the diagnostic image quality equivalent to current clinical standards. Various image reformatting techniques, including linear or curved multiplanar reformation, minimum intensity projection, maximum intensity projection, and volume rendering, are useful for processing depending upon the purpose [16].

## 3 SUB VALVULAR AORTIC STENOSIS

Subvalvular aortic stenosis (SAS) refers to LV outflow obstruction just under the aortic valve in the LVOT region. It can occur in isolation or in association with other CHDs. The prevalence of discrete SAS in adult patients with congenital heart disease is ≈6.5%, with a male-to-female ratio of 2:1 [17]. It has been postulated that SAS is caused by chronic flow disturbance, usually in a small and long outflow tract. The shear stress caused by abnormal flow patterns and chronic turbulence leads to the proliferation of abnormal endothelial substrate resulting in SAS [18]. The common presenting symptoms include angina, heart failure, or syncope. There are four different morphological types of SAS: (1) discrete fibromuscular, (2) tunnel type, (3) associated with the membranous ventricular septal defect, and (4) hypertrophic obstructive cardiomyopathy [19].

Discrete fibromuscular is the most common type of subaortic stenosis. It varies in morphology and can appear as a fibrous shelf or a ring below the aortic valve. The membrane typically extends from the interventricular septum around the margins of LVOT and falls short in the aortomitral region forming a horseshoe shape. This type of SAS is rare in infancy and is usually diagnosed after 3 years [20]. Tunnel type of SAS refers to a long segment fibromuscular tubular-shaped narrowing of LVOT. It is usually associated with other forms of LVOT obstruction like an interrupted aortic arch. SAS associated with membranous VSD occurs due to malalignment of the conal septum. Approximately 37% of patients with SAS may also have concomitant VSD of the perimembranous type. The posterior deviation of the conal septum leads to crowding and narrowing of LVOT resulting in SAS. This is in contrast to anterior deviation of conal septum which leads to RVOT stenosis in the Tetralogy of Fallot [21]. This form of SAS is also associated with interrupted aortic arch and coarctation of the aorta. Hypertrophic cardiomyopathy (HCM) is defined as LV hypertrophy that can’t be explained by heart or systemic disease. It is an autosomal dominant disease caused by mutations in the genes encoding cardiac sarcomeres proteins [22]. Hypertrophy can be concentric, apical, midventricular, or asymmetric septal. Out of these variants, the asymmetric septal type results in a sigmoidal contour of the septum which encroaches on the LVOT [23]. This pattern is associated with the systolic anterior motion of the mitral valve (SAM) resulting in subaortic obstruction. LVOT obstruction is provoked by physiological or pharmacological measures but it can be present at rest in approximately 30% of patients [24].

### 3.1 CT Assessment of SAS

The goals of imaging in a patient with SAS are to:

a. Identify the level of LVOT obstruction and distinguish between types of SAS

b. Define the degree of obstruction and secondary changes in LV

c. Delineate the relationship of the stenosis to key cardiac structures

d. Identify associated cardiac defects

**Fig. (1):**
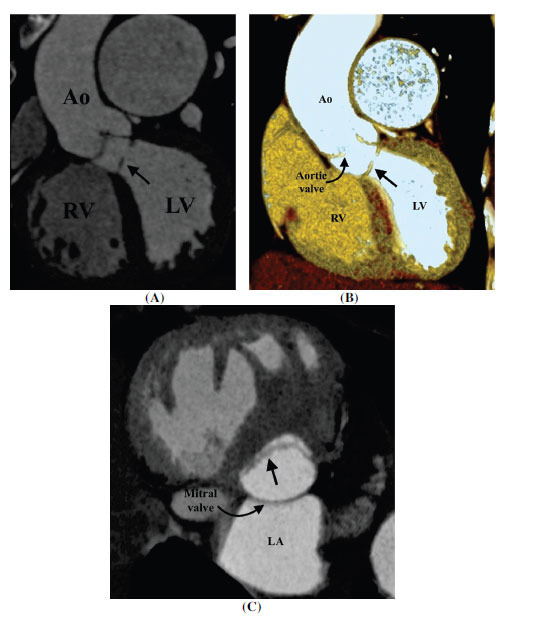
Membrane-type subaortic stenosis. (**A**) Coronal contrast-enhanced computed tomographic angiography image and (**B**) thin volume rendered technique image showing a shelf-like fibromuscular in the subaortic region (arrow). (**C**) Axial minimum intensity projection image showing the classical appearance of the subaortic membrane (arrow). It falls short in the aortomitral region forming a horseshoe shape. **Abbreviations:** RA; right atrium, RV; right ventricle, LV; left ventricle, Ao; ascending aorta.

**Fig. (2):**
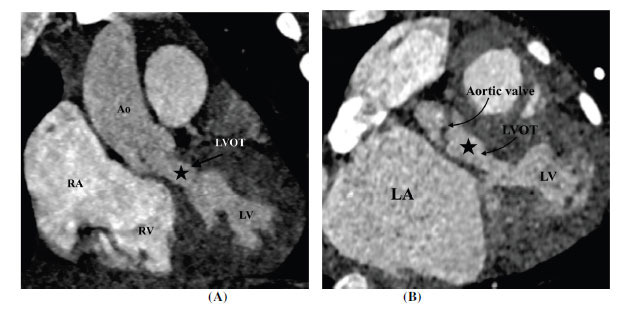
Tunnel type subaortic stenosis (**A**) Coronal contrast-enhanced computed tomographic angiography image and (**B**) oblique coronal contrast-enhanced computed tomographic angiography image showing tunnel-like subaortic stenosis (arrows). **Abbreviations:** RA; right atrium, RV; right ventricle, LV; left ventricle, Ao; ascending aorta.

**Fig. (3):**
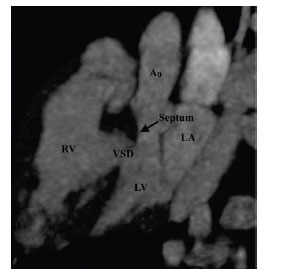
Subaortic stenosis associated with ventricular septal defect. Sagittal oblique contrast-enhanced computed tomographic angiography image showing the ventricular septal defect. There is associated posterior deviation of the infundibular septum (asterisk) causing LV outflow tract obstruction. **Abbreviations:** LA; left atrium, LV; left ventricle, RV; right ventricle, Ao; ascending aorta, VSD; ventricular septal defect.

**Fig. (4):**
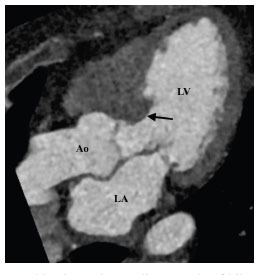
Subaortic stenosis associated with hypertrophic obstructive cardiomyopathy. Oblique contrast-enhanced computed tomographic angiography three-chamber view image showing asymmetrical basal septal hypertrophy (arrow). **Abbreviations:** LA; left atrium, LV; left ventricle, Ao; ascending aorta.

MDCT is helpful in delineating the morphology of LVOT and distinguishing the different types of SAS. The discrete fibromuscular SAS appears as shelf-like soft tissue thickening or a thin membrane-like structure in the subaortic region. The subaortic membrane usually forms a horseshoe morphology extending around the LVOT margins with a deficient component in the aortomitral region. Rarely, it forms a complete ring (Fig. **1**). Tunnel-type SAS appears as a thick long segment of narrowing in LVOT region (Fig. **2**). VSD related type shows a perimembranous type VSD with the posteriorly deviated conal septum (Fig. **3**). Both tunnel type and VSD related type SAS are associated with other obstructive lesions along the aorta like interrupted aortic arch and aortic coarctation. HCM-related SAS is characterized by asymmetric septal hypertrophy (Fig. **4**). The diagnostic criteria for HCM consist of a maximum LV wall thickness ≥15 mm or the ratio of the septal to the inferior wall thickness greater than 1.5 at the midventricular level. In children, septal wall thickness greater than or equal to two standard deviations (z score ≥2) is used as diagnostic criteria [22].

The severity of SAS is better estimated by echocardiography. Continuous wave Doppler measurement of maximal instantaneous and mean systolic gradients provides an estimate of the severity of LVOT stenosis. Guidelines suggest that a maximum instantaneous gradient of ≥50 mm Hg or mean gradient of ≥30 mm Hg across LVOT is an indication for surgical repair even in the absence of symptoms or adverse sequelae, such as aortic regurgitation [25]. CT has a limited role in estimating the severity of SAS; the assessment is subjective and limited to mild, moderate, and severe types without any precise definitions. Recently, the latest generation of MDCT has shown a promising role in the assessment of structural heart diseases. MDCT provides a three-dimensional volumetric data set of the entire heart that can be reconstructed at any point in the cardiac cycle providing a comprehensive assessment of cardiac structure and function. For example, in HCM, the systolic and diastolic phase images provided by MDCT provide exact measurements of LV wall thickness, distribution of myocardial hypertrophy, and identification of any apical aneurysm formation. Furthermore, using retrospective ECG triggering, SAM can be demonstrated on cine images. In addition, MDCT angiography can demonstrate the prominent septal perforators supplying the hypertrophied septal myocardium, which is important if any ablation procedure is being considered [26]. The secondary changes in SAS include LVH, LV dysfunction, and finally heart failure. Evaluation of LV volume, mass, and ejection fraction are important parameters that guide patient management. MDCT allows the evaluation of LV volume, mass, and ejection fraction. CT-derived EF has shown excellent agreement with two-dimensional echocardiography and magnetic resonance imaging, [27-30]. MDCT-derived LV mass has also shown high agreement with MR imaging measurements [31].

The third important goal of imaging is to delineate the relationship of the stenosis to key cardiac structures. Studies have shown that patients with VSD or coarctation of the aorta or both, who have exaggerated aortic override, wider mitral-aortic separation, and steeper aortoseptal angle subsequently develop subaortic stenosis [32-34]. Although the importance of these measurements has been evaluated with echocardiography mainly, CT can be a helpful adjunct in cases with unfavourable acoustic windows. It is important to accurately delineate the relationship and attachment of the subaortic membrane with the mitral valve apparatus. Most of the time the subaortic membrane is horseshoe shaped and falls short in the aortomitral region. Rarely, it may adhere to the anterior mitral leaflet resulting in an additional source of obstruction. Subaortic stenosis may also develop following tunnel repair of VSD in various congenital heart diseases. Any fibrous growth on the patch material may cause turbulent flow across the outflow tract resulting into a subaortic obstruction. In such cases, CT may aid in the anatomic assessment and operative planning [35].

SAS is associated with multiple other cardiac defects. The commonly associated lesions include the bicuspid aortic valve, coarctation of the aorta, and interrupted aortic arch. Shone complex is a rare syndrome consisting of multiple left-sided, LVOT obstructive lesions. It was described in 1963 and the classical Shone complex consists of a supravalvular mitral membrane, sub-valvular aortic stenosis, parachute mitral valve, and coarctation of the aorta [36]. Most patients have only a few of these obstructive lesions; commonly called incomplete Shone syndrome. MDCT using multiplanar reformations can delineate all the components of Shone complex.

### 3.2 Management of SAS

There is no established medical therapy or palliative procedures like balloon dilation for SAS. Surgical intervention is the appropriate treatment. The commonly preferred procedure is the surgical resection of the sub-valvular membrane with or without septal myectomy [37]. For patients with diffusely narrowed LVOTs and HCM, the Konno procedure and its modifications may be necessary, which involves aorto-ventriculoplasty with autografts. The common postoperative complication includes heart block, iatrogenic VSD, mitral valve injury, and incomplete resection or recurrence [38].

### 3.3 Valvular Aortic Stenosis

Valvular aortic stenosis is the most common type of LV outflow tract obstruction and accounts for 80% of all cases. The most common congenital cause of aortic valve stenosis is bicuspid aortic valve (BAV) with a prevalence of 1%-2% in the general population [39]. The definitive cause for the abnormal valve is not clear and likely multifactorial. Theories state that reduced flow across the aortic valve results in failure of leaflets separation. Theories have also postulated abnormal cellular migration and signalling pathways in abnormal aortic root development; resulting in annular hypoplasia, poorly developed valve commissures, and myxomatous thickened valvular leaflets [40].

**Fig. (5):**
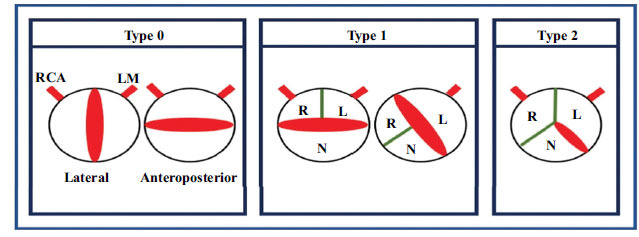
Schematic representation of various bicuspid aortic valve (BAV) morphologies according to the Sievers classification. The aortic valve is depicted on a cross-sectional short-axis view. The origin of the right coronary artery (RCA) and the left main (LM) are demarcated with red lines. Type 0: BAV without raphe, with lateral or anteroposterior orientation of the free edge of the cusps. Type 1: BAV with 1 raphe (in green), the most frequent type with the fusion of the right (R) and left (L) coronary cusps followed by fusion of the right and noncoronary (N) cusps. Type 2: BAV with 2 raphes.

There is marked heterogenicity in the phenotypes of BAV. Various classifications have been proposed by different authors to categorise BAV into different types based on the number of raphes and the spatial position of cusps or raphes. Sievers classification is the most commonly used in clinical practice, according to which BAV is divided into three categories: type 0, type 1, and type 2 (Fig. **5**). Type 0 is a true BAV with no raphe and two coronary cusps. It is further divided into lateral or anteroposterior (AP) categories depending upon the spatial position of cusps. The incidence of a type 0 phenotype in the original Sievers and Schmidtke series was found to be 7%. The Sievers type 1 BAV has a single raphe and two valve cusps. It is further divided into three subtypes depending on the spatial position of a single raphe: left-right subtype (L-R) with the fusion of right and left coronary cusps, right-non subtype (R-N) with the fusion of right and non-coronary cusps and non-left subtype (N-L) with the fusion of non-coronary and left coronary cusps. The incidence of type 1 phenotype was found to be 88%. The Sievers type 2 BAV has two raphes and two cusps. In the original series, the first raphe was identified between right and left coronary cusps, and the second raphe was seen between right and non-coronary cusps, classified as L-R/R-N. The incidence of type 2 was found to be 5%. It is imaginable that additional subcategories like the size of cusps, extension, and size of raphes, annulus dilatation, the concomitant existence of an ascending aortic aneurysm, and heritability could even more precisely specify the valve. However, the addition of these subcategories would make the classification system more complex and probably less practical [41].

BAV is associated with valvular stenosis, regurgitation, endocarditis, ascending aortic aneurysm, and aortic dissection [40]. Patients with BAV have an associated aortopathy which is primarily due to abnormal aortic substrate and not secondary to aortic stenosis. The aortopathy is similar to that seen in connective tissue disorders like Marfan syndrome and with an increased predisposition for aortic aneurysm, dissection, and rupture. Kang *et al.* investigated the association between BAV phenotypes and the pattern of valvular dysfunction or bicuspid aortopathy, using MDCT. They divided the bicuspid aortopathy phenotypes into three patterns according to the type of aortic dilation: type 1 (aortic enlargement confined to the sinus of Valsalva), type 2 (aortic enlargement involving the tubular portion of the ascending aorta), and type 3 (aortic enlargement extending to the transverse aortic arch). They divided the BAV phenotypes into two main categories: BAV-RL having a right-left orientation of free edges of cusps, and BAV-AP having anterior-posterior orientation of free edges of cusps. The subtypes in the BAV-RL phenotype included Sievers type 0 RL, type 1 R-N, and type 1 L-N; while subtypes in BAV-AP included Sievers type 0 AP and type 1 L-R. They demonstrated that the BAV-RL subtype is associated with moderate-to-severe aortic stenosis, while the BAV-AP subtype is associated with moderate-to-severe aortic regurgitation. A normal aorta was the most common phenotype in BAV-AP patients, and type 3 aortopathy was the most common phenotype in BAV-RL patients. The study concluded that there is a meaningful association between BAV phenotypes and the various types of valvular dysfunction or aortopathy [42]. Although they couldn’t explain why a specific BAV phenotype is associated with a certain type of valvular dysfunction and aortopathy, a recent animal experiment study has provided strong evidence that BAV-AP and BAV-RL are distinct etiological entities. The authors clearly demonstrated that BAV-AP results from the anomalous septation of the embryonic outflow tract while BAV-RL reflects the defective development of endocardial cushions [43]. Recent guidelines suggest that patients with aortic aneurysms associated with BAV should undergo elective operation at smaller diameters (4.0 to 5.0 cm depending on the condition) instead of 5.5cm, which is the normal recommended threshold in the general population [44]. The incidence of infective endocarditis (IE) is also disproportionally high in patients with BAV. Studies have shown that 25%-54% of all infected aortic valves are bicuspid [45-46].

BAV is also known to coexist with other congenital cardiovascular defects like coarctation of the aorta [47], atrial septal defect, and ventricular septal defect [48]. A number of syndromes involving left-sided obstructive lesions have associated BAV: hypoplastic left heart syndrome with hypoplastic LV, mitral atresia, aortic stenosis, and coarctation of the aorta [49]; Shone syndrome with supravalvular mitral membrane, sub valvular aortic stenosis, parachute mitral valve and coarctation of the aorta [50]; William syndrome with supravalvular aortic stenosis, pulmonary artery stenosis, coronary ostial stenosis, and systemic arterial stenosis [51]; and Turners syndrome with hypoplastic left heart, aortic stenosis, and coarctation of the aorta [52]. Finally, some reports have suggested the reversal of coronary dominance in BAV patients with an unusually high incidence of left dominance [53].

### 3.4 CT Assessment of Valvular Aortic Stenosis

The goals of imaging in a patient with valvular aortic stenosis are to:

a. Define the valve morphology

b. Assess the degree of stenosis and secondary changes in LV

c. Identify the complications and associated lesions

**Fig. (6):**
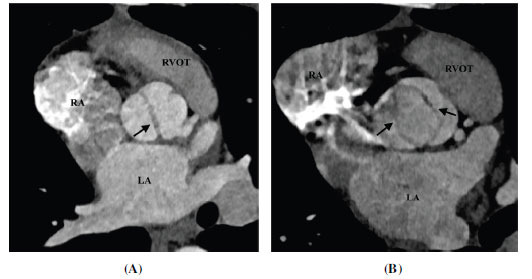
Type 0 (AP) bicuspid aortic valve. (**A**) Axial contrast-enhanced computed tomographic angiography image diastolic frame and (**B**) Axial contrast-enhanced computed tomographic angiography image systolic frame image showing a bi-commissural non raphe type 0 bicuspid aortic valve (arrows). The free cusps are oriented in the anteroposterior direction (AP subtype). **Abbreviations:** RA; right atrium, RVOT; right ventricular outflow tract, LA; left atrium, LV; left ventricle, Ao; ascending aorta.

**Fig. (7):**
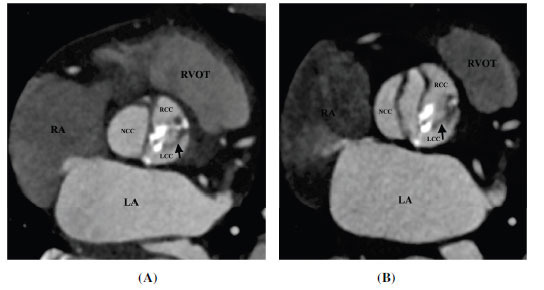
Type 1 (R-L) bicuspid aortic valve. (**A**) Axial contrast-enhanced computed tomographic angiography image diastolic frame and (**B**) Axial contrast-enhanced computed tomographic angiography image systolic frame image showing bicuspid aortic valve with a thick calcified raphe (arrow) between right and left coronary cusps (R-L subtype). **Abbreviations:** RA; right atrium, RVOT; right ventricular outflow tract, LA; left atrium, LV; left ventricle, Ao; ascending aorta, RCC; right coronary cusp, LCC; left coronary cusp, NCC; non-coronary cusp.

**Fig. (8):**
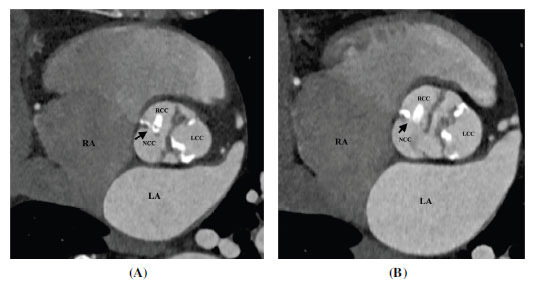
Type 1 (R-N) bicuspid aortic valve. (**A**) Axial contrast-enhanced computed tomographic angiography image diastolic frame and (**B**) Axial contrast-enhanced computed tomographic angiography image systolic frame image showing bicuspid aortic valve with a thick calcified raphe between right and non-coronary cusps (R-N subtype). **Abbreviations:** RA; right atrium, RVOT; right ventricular outflow tract, LA; left atrium, LV; left ventricle, Ao; ascending aorta, RCC; right coronary cusp, LCC; left coronary cusp, NCC; non-coronary cusp.

**Fig. (9):**
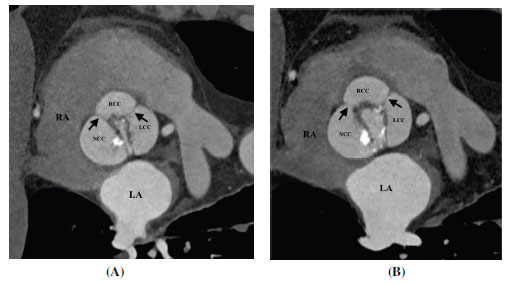
Type 2 bicuspid aortic valve. (**A**) Axial contrast-enhanced computed tomographic angiography image diastolic frame and (**B**) Axial contrast-enhanced computed tomographic angiography image systolic frame image showing bicuspid aortic valve with a two thin non-calcified raphe between right-left and right- non-coronary cusps. **Abbreviations:** RA; right atrium, RVOT; right ventricular outflow tract, LA; left atrium, LV; left ventricle, Ao; ascending aorta, RCC; right coronary cusp, LCC; left coronary cusp, NCC; non-coronary cusp.

TTE is generally the first modality to evaluate BAV. However, the technical issues related to body habitus and heavy leaflets calcification may limit the valve evaluation; the determination of the number and arrangement of cusps may be unclear in up to 25% of patients [54]. CT provides a more accurate assessment of BAV (Figs. **6-9**) . Tanaka *et al.* conducted a study to evaluate the diagnostic value of CT for the evaluation of BAV. The sensitivity, specificity, positive predictive value, and negative predictive value for the detection of a BAV were 76.5%, 60.6%, 68.4%, and 95.2%, respectively for TTE and 94.1%, 100%, 100%, and 97.1%, respectively for CT. CT was found superior to TTE especially in patients with extensively calcified aortic valves [55]. CT allows a clear depiction of cusps, commissures, raphes, and leaflet calcifications. BAV morphology can be classified using the Sievers system. It is challenging to differentiate bicuspid aortic valves with raphe from tricuspid aortic valves due to their similar appearance, particularly during diastole, as the raphe can be mistaken as a normal commissure. However, the simultaneous assessment of both diastolic and systolic data sets can reliably differentiate between these two types.

**Fig. (10):**
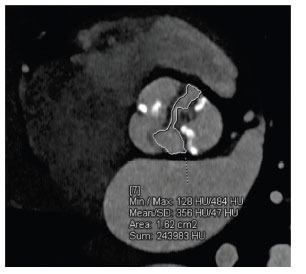
Axial contrast-enhanced computed tomographic angiography image showing the planimetry of the bicuspid aortic valve.

CT is also an excellent modality to assess the various valvular and aortic complications associated with BAV. Aortic stenosis (AS) is the most common complication of BAV. The leaflets of BAV undergo thickening, fibrosis, and calcification early in life that progresses after the fourth decade. These patients eventually need valve replacement in the sixth or seventh decade of life. CT is an excellent modality to assess the aortic valve structure, area, and motion. CT is superior to TEE and MRI in identifying and characterizing valvular calcification. The calcification is usually confined to the raphe and base of cojoined cusps. The valvular calcification can be scored similarly to coronary calcium. CT-derived calcium score is highly reproducible and demonstrates excellent discrimination for detecting severe AS [56]. Recent work suggests that an Agatston score of 1651 provides the sum of sensitivity plus specificity in accurately differentiating severe and non-severe aortic stenosis [57]. CT-based planimetry can be used to measure the aortic valve area (AVA). CT-derived planimetric measurements (Fig. **10**) are highly reproducible and correlate with TTE and MR planimetric measurements. CT facilitates the accurate identification of patients having moderate or severe aortic stenosis with sensitivity, specificity, and diagnostic accuracy values approaching 100% [58].

Aortic stenosis is usually associated with aortic regurgitation (AR) also. AR is caused by fibrous retraction or prolapse of cusps, valvular destruction due to infective endocarditis, or due to aneurysmal dilatation of the aortic root and annulus. BAV is the most common cause of aortic regurgitation necessitating valve replacement. CT can also provide useful information on AR, especially in patients with poor acoustic windows on TTE and contraindications to CMR. Feuchtner *et al.* compared the diagnostic accuracy of 64-slice MDCT with TTE in the evaluation of AR. The sensitivity, specificity, PPV, and NPV of CT for the detection of moderate and severe aortic valve regurgitation were 95%, 100%, 100%, and 98%, respectively. CT-derived aortic regurgitant orifice area correlated significantly with the severity of aortic valve regurgitation by TTE. The study concluded that 64-MDCT can accurately detect moderate to severe aortic regurgitation; however mild aortic regurgitation may be missed especially in the presence of severe valve calcification [59]. Furthermore, CT has also demonstrated excellent agreement in assessing the mechanisms of AR, *i.e.* aortic root dilatation (type I), cusp prolapse (type II), and restrictive cusp motion (type III), using surgical inspection as a reference. A study conducted by Goffinet *et al.* showed that ARA measured by both MDCT and MR allows accurate quantitative assessment of AR. Both techniques can also accurately determine the mechanism of AR [60].

BAV is frequently complicated by IE. The incidence of IE in the BAV population ranges from 10% to 30%; approximately 25% of IE occurs in patients with BAV [61, 62]. The patients with BAV-related IE are younger than with those having tricuspid valves and they have fewer comorbidities. There is a higher tendency of staphylococcal origin and more predilection for peri-valvular complications, in particular, abscesses [63]. Other commonly seen perivalvular complications include pseudoaneurysm, and aortocavitary fistula [64]. Cardiac CT has a complementary role to TTE in the workup of these patients especially in cases with leaflets calcifications and prosthetic valves [65]. CT has been incorporated into the 2015 European Society of Cardiology modified diagnostic criteria for IE [66]. CT provides useful information about valvular and perivalvular structures. CT-derived 2D MPR and 3D reconstruction provide a better understanding of vegetation and other intracardiac complications. Gurdun *et al.* reported comparable diagnostic performance of CT in detecting evident valvular abnormalities for IE compared with TEE with 97% sensitivity and 88% specificity. CT correctly identified 96% of patients with valvular vegetations and 100% of patients with abscesses/pseudoaneurysms in comparison with the intraoperative specimen. The diagnostic accuracy for the detection of vegetations and abscesses/pseudoaneurysms didn’t show any significant differences as compared with TEE [67].

**Fig. (11):**
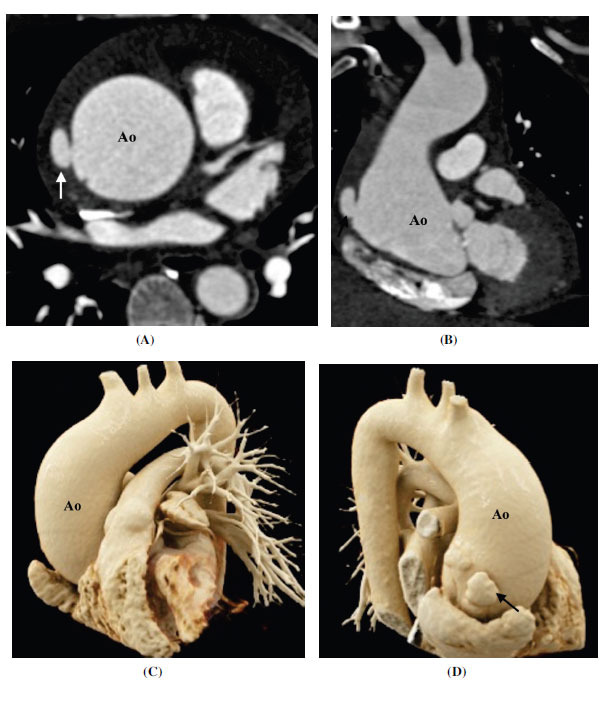
Type 2 bicuspid aortopathy. (**A**) Axial and (**B**) coronal contrast-enhanced computed tomographic angiography image showing dilated ascending aorta. A small outpouching suggesting a pseudoaneurysm is seen arising from the right lateral aortic wall. (**C**) Frontal and (**D**) back cinematic rendered image showing dilated ascending aorta and pseudoaneurysm. **Abbreviation:** Ao; ascending aorta.

Patients with BAV are also at risk of various aortic complications like aortic dilatation, aneurysm, and dissection. Currently, aortopathy is considered to be the underline mechanism for aortic dilatation (Fig. **11**). The BAV aortopathy is distinct from TAV aortopathy in terms of epidemiology, macroscopy, and microscopic features. CT angiography allows thorough evaluation of the aorta with precise aortic measurements using MPR images. Due to cardiac pulsation and aortic root motion, the ECG-gated scan provides the best results for evaluating the aortic root and ascending aorta. The measurements obtained using non-gated scans may overestimate aortic diameter leading to inappropriate surgery referrals. Irrespective of the prospective or retrospective technique, gating improves the accuracy and reproducibility of measurements [68, 69].

### 3.5 Management of Valvular Aortic Stenosis

The management of valvular aortic stenosis is determined by the age of the patient at presentation and the severity of the valvular obstruction. The current therapeutic options are percutaneous balloon aortic valvuloplasty, and surgical aortic valvotomy or valve replacement.

Both balloon valvuloplasty and surgical valvotomy are firmly established as initial treatment methods with an ongoing debate about the best one during childhood. However, there are also groups that advocate early valve replacement as the best intervention, but no randomized series for different treatment strategies exists [70].

### 3.6 Supravalvular Stenosis

Supravalvular aortic stenosis (SVAS) is a rare congenital anomaly with variable degrees of LV outflow obstruction distal to the aortic valve. It accounts for 8 -14% of all cases of congenital aortic stenosis. Histologically it is characterized by intima-medial fibrosis accompanied by smooth cell hypertrophy and increased collagen deposition. These findings result in increased shear stress and reduced elasticity. There are three morphological subtypes of SVAS: membranous, hourglass, and diffuse. The hourglass type of SVAS is the most common followed by the membranous type. The diffuse type is rare with challenging surgical repair due to the variable length of aortic hypoplasia [71]. All three types are associated with a mutation in the elastin gene on chromosome 7q11.23. SVAS may present as sporadic cases with normal faces and intelligence; autosomal dominant with normal faces and intelligence; or Williams-Beuren syndrome with abnormal faces and mental retardation [72].

SVAS is associated with multiple other abnormalities of the LV outflow tract. Aortic valve abnormalities are seen in 50% of the patients; the most common being a BAV [73]. This is followed by SAS, which occurs in 16% of the cases and contributes to aortic valve damage [74]. The patient's symptomatology and long-term outcome are determined by multiple factors. The poorly distensible sinotubular junction increases the shear forces and fatigue stress on aortic leaflets resulting into leaflets thickening and sclerosis [75]. The resultant aortic stenosis, regurgitation, or both are the most common etiologies for frequent surgeries [74, 76]. The patients with SVAS are also at high risk of myocardial ischemia which occurs due to impaired filling of coronary arteries and intramyocardial perfusion mismatch. The varying degree of aortic leaflet adhesion to the narrowed STJ hampers the diastolic phase filling of coronary arteries [77, 78]. Furthermore, the coronary arteries are exposed to high systolic pressure in SVAS, which results into coronary dilatation, tortuosity, and premature atherosclerosis. This is further compounded by increased myocardial mass and intramyocardial pressure resulting into perfusion mismatch [79]. These mechanisms play an important role in the patient symptomatology and long-term outcome of patients with SVAS.

### 3.7 CT assessment of Supravalvular Aortic Stenosis

The goals of imaging in a patient with SVAS are:

a. Delineation of the site and morphology of the stenosis

b. Concomitant evaluation of ascending aorta, coronary arteries, and head-and-neck vessels.

c. Assessment of the secondary changes in LV

d. Identification of the associated LVOT obstructive lesions

**Fig. (12):**
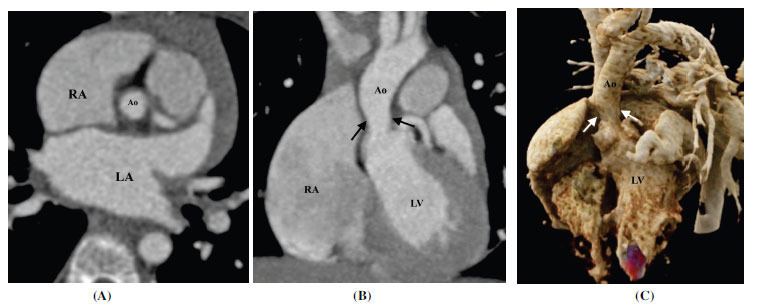
Supravalvular aortic stenosis. (**A**) Axial contrast-enhanced computed tomographic angiography image showing the narrowed caliber of the supravalvular aorta (**B**) Coronal contrast-enhanced computed tomographic angiography image and (**C**) Volume rendered technique image showing supravalvular aortic stenosis (arrow). **Abbreviations:** RA; right atrium, LA; left atrium, LV; left ventricle, Ao; ascending aorta.

**Fig. (13):**
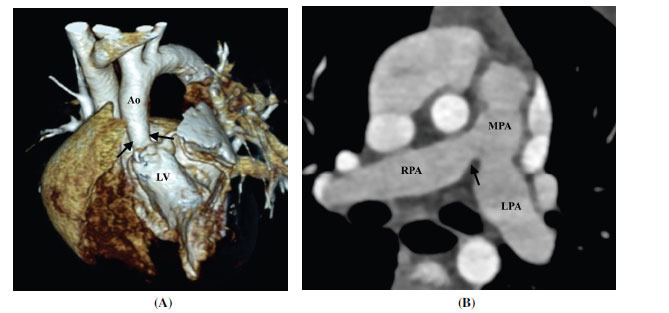
Supravalvular aortic stenosis in a case of William syndrome. (**A**) Volume rendered technique image showing supravalvular aortic stenosis (arrow) (**B**) Axial contrast-enhanced computed tomographic angiography image showing mild focal stenosis at the ostium of the right pulmonary artery (arrow).

CT can delineate the site and morphology of different types of SVAS with high accuracy (Fig. **12**). The membranous type SVAS appears as a shelf-like soft tissue thickening or a thin membrane-like structure in the supravalvular region. The hourglass type is characterized by the narrowing of the sinotubular junction with normal or mildly dilated ascending aorta producing hourglass-shaped appearance. The diffuse type of SVAS is characterized by generalized hypoplasia of ascending aorta which may involve the aortic arch as well. CT is also helpful in assessing the aortic root, coronary arteries, arch vessels, and pulmonary arteries. Using the retrospective gating protocol, the ventricular size and function can also be assessed simultaneously to decide future surgical planning. In addition, if the patient has Williams syndrome, imaging of the entire thoracoabdominal aorta can delineate the presence of arterial stenosis at different levels (Fig. **13**). Furthermore, CT can assess the other associated LV outflow obstructive lesions including SAS, BAV, and coarctation of the aorta in a single study [80, 81].

### 3.8 Management of SVAS

Surgical correction of the SVAS is indicated in symptomatic patients (angina, syncope, and dyspnea) or with patients having a mean pressure gradient ≥ 50mm of Hg. The current surgical strategies focus on preserving root morphology and salvaging the native aortic valve, if possible. The localized type of SVAS is corrected by excision of the supravalvular ridge and by augmenting all three sinuses. The native aortic valve is salvaged by augmentation, commissuroplasty, annuloplasty, or thinning. The Ross procedure is a newer option, for patients requiring replacement of aortic valve. The diffuse form of SVAS is more difficult to treat. The various surgical techniques include (1) the insertion of an LV to apicoaortic conduit, (2) Extensive endarterectomy of the entire ascending aorta, followed by augmentation using dacron patch augmentation (3) Insertion of an interposition graft if the ascending aorta is severely hypoplastic. Patients with Williams syndrome and SVAS show spontaneous regression of stenosis without intervention, and overall patient survival is excellent after treatment (94% at 10 years) [75, 76, 82].

### 3.9 Coarctation of Aorta

Aortic coarctation (AC) accounts for 6-8% of congenital heart defects. Male infants are affected more commonly, with a higher incidence in stillborn infants [83]. Several hypotheses have been proposed to explain the embryology of AC. The common explanations include the extension of contractile ductus arteriosus tissue into the aortic wall resulting into luminal obliteration; and reduced anterograde blood flow through the aortic valve leading to isthmic site stenosis [84]. However, these singular mechanistic views do not represent the whole underlying process, considering the widespread association of aortic coarctation with other associated congenital abnormalities in the heart and in intracranial vasculature, such as cerebral aneurysms [83]. Most of the cases are sporadic; however, there is a genetic component also [85]. There is a frequent association of AC with Turner syndrome [86]. AC is also associated with other congenital heart defects. The bicuspid aortic valve is the most commonly associated abnormality, reported in up to 75% of patients with AC [87, 88]. The other associated cardiac defects include aortic arch hypoplasia, PDA, and mitral valve abnormalities [83, 89].

AC shows three different phenotypes depending upon the site of aortic narrowing in relation to the ductus insertion: proximal (preductal), opposite (juxtaductal), or immediately distal (postductal). Classically, two different forms of AC have been described: infantile and adult. The infantile type is characterized by preductal constriction. It is commonly associated with other cardiac defects and various syndromes. The adult type is characterized by juxtaductal or postductal constriction, with rare atypical narrowing in the abdominal aorta in a few cases. The descending aortic perfusion is dependent on PDA in the preductal type and on collateral vessels in the postductal type [90, 91]. The clinical manifestations depend on the location and degree of luminal narrowing; extent of the collateral circulation; and associated congenital heart defects. The infantile or preductal AC presents with early neonatal acute heart failure; while the adult type presents either incidentally, or with treatment resistive severe hypertension. The classic sign of AC is the disparity in pulsation and blood pressure in the arms and legs, being lower in the latter. In the adult form- juxtaductal or post-ductal type, the AC is associated with a closed ductus arteriosus. The constriction is usually located in the post-ductal aorta. Atypical locations are possible, like in the abdominal aorta. In the latter location, it is essential to search for an extension to the renal arteries. Adults with postductal coarctation usually remain asymptomatic and are diagnosed by a physical examination that reveals systemic arterial hypertension in the arms with diminished femoral arterial pulse [92, 93].

The complications of AC are related to systemic hypertension, including premature coronary artery disease, heart failure, hypertensive encephalopathy, or intracranial haemorrhage from rupture of the congenital circle of Willis aneurysm. Infective endocarditis or endarteritis is a significant complication in adults. Aneurysms of the descending aorta or collateral vessels are also seen [94].

### 3.10 CT Assessment of Aortic Coarctation

The goals of imaging in a patient with AC are:

a. Delineation of the site and morphology of the AC.

b. Localization of the collaterals pathways.

c. Assessment of the secondary changes in LV

d. Identification of the associated LVOT obstructive lesions

e. Evaluation of postoperative or postprocedural graft anastomoses or stents.

**Fig. (14):**
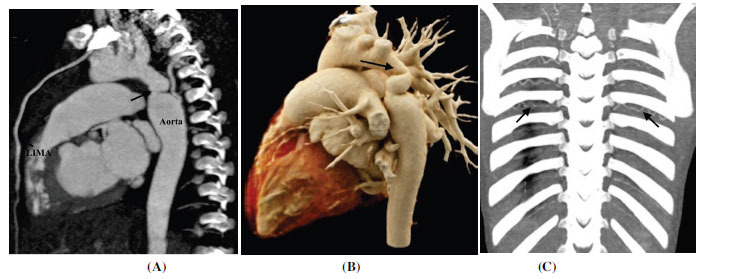
Post ductal aortic coarctation. (**A**) Sagittal contrast-enhanced computed tomographic angiography image showing focal narrowing of the aortic lumen (arrow) distal to the origin of the left subclavian artery. The left internal mammary artery is enlarged to provide collateral circulation to descending thoracic aorta (**B**) Volume rendered technique image confirms the findings. (**C**) Sagittal and (E) Coronal contrast-enhanced maximum intensity projection images showing collateral circulation (arrows) through enlarged left internal mammary retry and intercostal arteries respectively. **Abbreviation:** LIMA; left internal mammary artery.

**Fig. (15):**
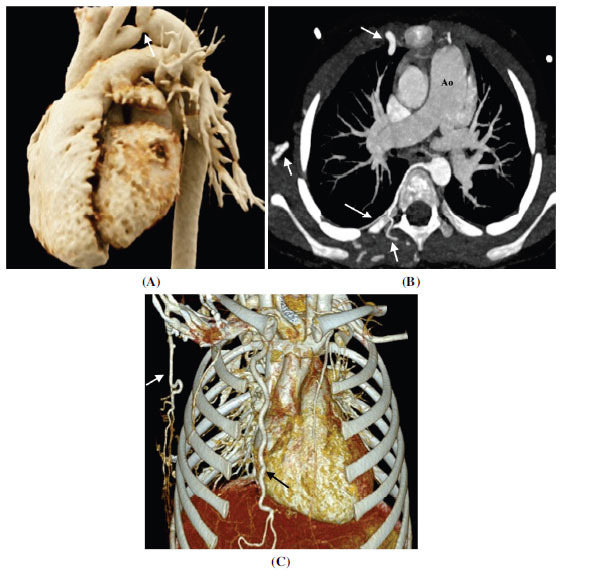
Preductal aortic coarctation. (**A**) Volume rendered technique image showing focal narrowing of the aortic lumen at the left subclavian artery origin and just proximal to it. (**B**) Axial contrast-enhanced computed tomographic angiography image and (**C**) Volume rendered technique image showing enlarged rights subscapular, right internal mammary, and posterior intercostal arteries providing collateral circulation to descending thoracic aorta. Note that the collateral circulation is provided through the ride side only as the aortic narrowing is proximal to the left subclavian artery.

**Fig. (16):**
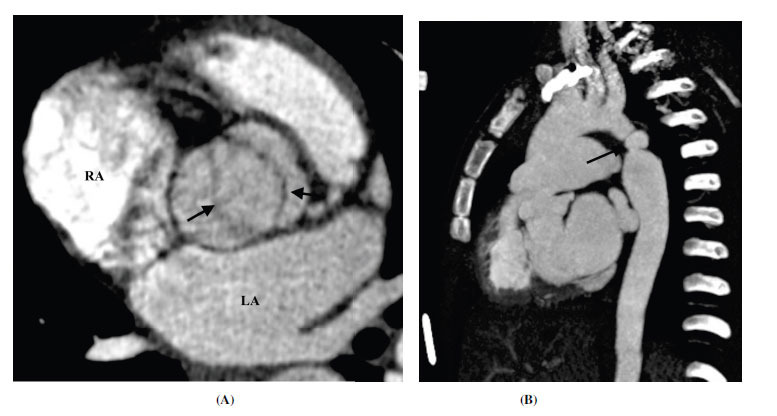
Post ductal aortic coarctation in a case of Turner syndrome. (**A**) Axial contrast-enhanced computed tomographic angiography image showing bicuspid aortic valve. (**B**) Sagittal contrast-enhanced computed tomographic angiography image showing focal narrowing of the aortic lumen distal to the origin of the left subclavian artery (arrow).

**Fig. (17):**
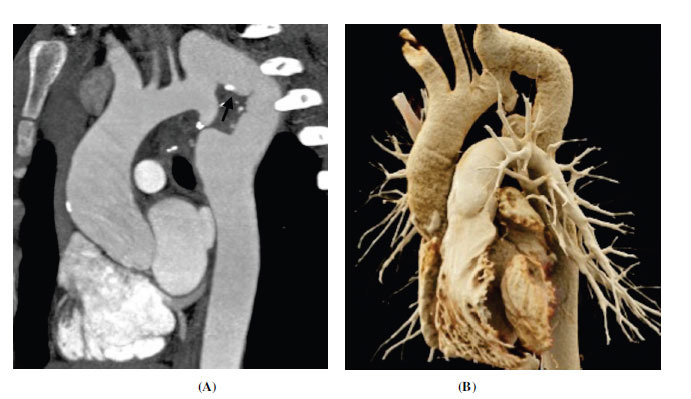
Aortic pseudo coarctation (**A**) Sagittal contrast-enhanced computed tomographic angiography image and (**B**) Cinematic rendered image tortuosity of the aortic arch at the level of isthmus. No collateral vessels are seen. Echocardiography showed no gradient across the tortuous segment suggesting pseudo coarctation.

MDCT with three-dimensional and multiplanar reconstruction techniques provide state-of-the-art images of AC and its relationship to the arch vessels. The isotropic reformatted images created in different planes provide excellent anatomic detail of the aorta and the coarctation segment (Figs. **14-16**). MDCT is also helpful in demonstrating the various collateral circulatory pathways from internal mammary arteries, intercostal arteries, thyrocervical trunk, thoracoacromial trunk, and descending scapular arteries. The collateral vascularity can be analyzed in detail, which is an indirect clue to the severity of the stenosis. MDCT also provides reliable information about the biventricular function, although such information preferably should be acquired with echocardiography or MRI to reduce the radiation dose. CT is also helpful in identifying the associated cardiac defects like BAV, hypoplastic arch, and PDA. The dimensions of the aortic root and ascending aorta should also be included in the report for further follow-up imaging. It is important to distinguish AC from pseudo-aortic coarctation. Pseudo-coarctation is a normal variant defined by an excessive length of the aortic arch and the first portion of the descending aorta resulting in the folding or “kinking” effect next to the arterial ligament. The characteristic appearance along with the lack of collateral channels is helpful in the confident diagnosis of pseudo-coarctation (Fig. **17**) [95].

**Fig. (18):**
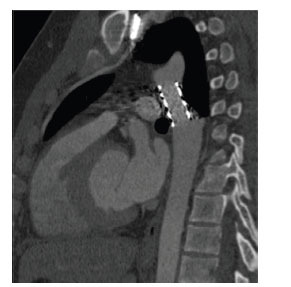
Aortic coarctation post stent placement. Sagittal contrast-enhanced computed tomographic angiography image showing metallic stent at the site of previous aortic narrowing. The stent lumen appears patent. The stent integrity is also maintained.

**Fig. (19):**
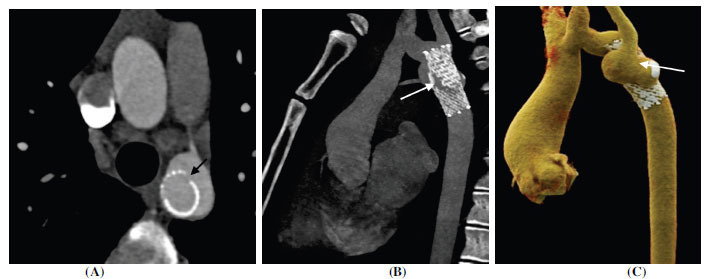
Pseudoaneurysm in aortic coarctation post stent placement. (**A**) Axial contrast-enhanced computed tomographic angiography image showing metallic stent at the site of previous aortic narrowing. The stent lumen appears patent. The stent integrity is lost at focal places (arrow) with contained leakage of contrast on the anterior and lateral aspects. (**B**) Sagittal contrast-enhanced computed tomographic angiography image and (**C**) Volume rendered technique images showing loss of stent integrity with pseudoaneurysm formation around it.

**Fig. (20):**
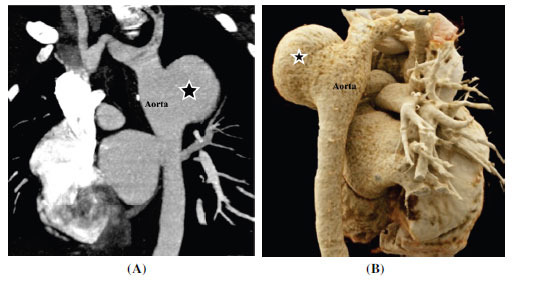
Saccular aneurysm in aortic coarctation post-surgical repair. (**A**) Sagittal contrast-enhanced computed tomographic angiography image showing broad neck saccular outpouching (*) of the proximal descending aorta at the site of repair of the coarctated segment. (**B**) Volume-rendered technique images from the back side confirm the findings.

MDCT is also useful for postoperative/postprocedural follow-up. CT can detect long-term complications such as intimal hyperplasia, in-stent stenosis, stent displacement, and aneurysm formation [96] (Figs. **18** and **19**). According to European guidelines, follow-up aortic imaging should be performed to document post-implantation anatomy and detect possible complications, with the ideal imaging interval depending on the exact baseline pathology [97]. American guidelines recommend follow-up with CT or MRI at intervals of five years or less after stent placement. CT is often the modality of choice since in-stent assessment with MRI can be hampered by artefacts [98]. The common complications after surgical repair include recurrent or residual aortic coarctation, and anastomotic site aneurysm formation [99] (Fig. **20**).

### 3.11 Management of AC

The American College of Cardiology and the American heart association recommend intervention in the following conditions: (1) Peak to peak Coarctation gradient > or equal to 20 mm Hg, (2) peak-to-peak coarctation gradient < 20 mm Hg with imaging evidence of significant coarctation significant collateral flow [100]. Surgical repair is the primary management. The surgical methods include resection of the coarcted segment followed by end-to-end anastomosis, patch aortoplasty, subclavian flap aortoplasty, and bypass graft across the coarctation if the length of the coarctated segment is very long. Endovascular treatment including balloon angioplasty and stent placement are also gaining acceptance. The main aim of this approach is to decrease the gradient across the coarctation site in a minimally invasive fashion avoiding the risk of surgery. The decision to perform endovascular versus surgical repair is made on a case-by-case basis depending upon the length of coarctation, vessel tortuosity, and arch hypoplasia [101].

## CONCLUSION

LVOT obstructions comprise a constellation of afterload-inducing lesions, with ventricular consequences ranging from LV hypertrophy to LV failure. CT can accurately and systematically delineate the various morphologic forms of LVOT obstruction. Current-generation scanners have significantly reduced the risk of radiation and contrast nephrotoxicity. CT techniques are evolving continuously and it promises to be a great helpful tool in preoperative planning and postoperative follow-up of CHD.

## LIST OF ABBREVIATIONS

CT: Computed Tomography
CMR: Cardiovascular Magnetic Resonance
LVOT: Left Ventricular Outflow Tract
LV: Left Ventricular
TTE: Transthoracic Echocardiography
CTA: Computed Tomographic Angiography
PC: Phase Contrast
MDCT: Multi-detector Computed Tomography
CHD: Congenital Heart Disease
SAS: Subvalvular Aortic Stenosis
